# SirA, CsrBC and HilD form in vivo a regulatory cascade that controls the SP1-1 and SPI-2 gene expression when *Salmonella* Typhimurium is in the intestinal lumen and are required for cecal colonization and liver dissemination in the avian model

**DOI:** 10.1007/s00203-025-04305-3

**Published:** 2025-04-02

**Authors:** José de Jesús Gómez-Chávez, Jwerlly Tatiana Pico-Rodríguez, Mireya Juárez-Ramírez, Hugo Martínez-Jarquín, Luary C. Martínez-Chavarría

**Affiliations:** Departamento de Patología, Facultad de Medicina Veterinaria y Zootecnia, Universidad Nacional Autónoma de México, Coyoacán, Ciudad de México, 04510 México

**Keywords:** *Salmonella*, SPI-1, SPI-2, Chicken, SirA, HilD

## Abstract

When *Salmonella* Typhimurium grows in LB in vitro, BarA/SirA system induces the expression of CsrB/C, that sequester the regulator CsrA, thus derepressing HilD regulator. HilD activated induces HilA and SsrB expression, central regulators of SPI-1 and SPI-2, respectively. We analyze the in vivo contribution of these genes in 1-day- and 1-week-old chickens infected with a Wild Type strain of *S.* Typhimurium and the Δ*sirA*, Δ*csrB/C* and Δ*hilD* mutants. CFUs determination in liver and cecum showed that the mutants colonized both organs in lower amounts compared with WT strain in both chicken models and they were affected in the ability to produce histological injuries in these organs. We analyzed whether these genes operate in cascade in vivo and prior to intestinal invasion, by analyzing *hilA*, *ssrAB*, *hilD*, *csrB* and *sirA* expression in the cecal contents of chickens inoculated with Wild Type and mutants 120 min after inoculation. Expression of *hilA* and *ssrB*, but not *csrB* and *sirA*, was decreased in Δ*hilD* strain. Expression of *hilD*, *hilA* and *ssrB*, but not *sirA*, was decreased in samples of Δ*csrB/C*. In SirA absence, expression of all genes was decreased. Our findings demonstrate that SirA, CsrB/C and HilD conform a regulatory cascade in vivo, when *Salmonella* is in intestinal lumen and this cascade controls the expression of HilA and SsrB prior to intestinal invasion. We also demonstrate that these genes are necessary for the production of lesions during *S*. Typhimurium infection in chickens.

## Introduction

*Salmonella* Typhimurium is a foodborne pathogen widely distributed in the world that infects a variety of mammals and birds (Andino and Hanning 2015; Lamas et al. 2018). The infection is characterized by generally self-limiting, gastroenteritis or severe systemic disease in some cases (Wotzka et al. 2017). Many virulence determinants of *S.* Typhimurium are located in regions of the genome known as *Salmonella P*athogenicity *I*slands (SPIs) (Gerlach and Hensel 2007). At least 23 pathogenicity islands have been discovered in the different strains of *Salmonella*, mainly *S.* Typhi and *S.* Typhimurium (Lu et al. 2022). The most deeply studied islands are SPI-1 and SPI-2 (Gerlach and Hensel 2007). Both contain genes that encode a variety of virulence factors such as a type 3 secretion system (T3SS), effector proteins, chaperones, and their own regulatory proteins (Bai et al. 2018).

SPI-1 contributes to host epithelial cell invasion, whereas SPI-2 allows bacterial survival within cells of a wide variety of hosts (Ilyas et al. 2017), but it has been revealed that SPI-2 genes also play a role in the development of the intestinal inflammatory disease and, accordingly, they are expressed in the intestinal lumen (Bispham et al. 2001; Brown et al. 2005; Coburn et al. 2005; Coombes et al. 2005; Hapfelmeier et al. 2005; Jones et al. 2007).

The expression of both islands is highly controlled by proteins encoded inside and outside of the islands. Usually, expression of both islands is repressed by H-NS, a negative global regulator that represses *hilA* and *ssrAB*, central regulators of SPI-1 and SPI-2, respectively (Lucchini et al. 2006; Navarre et al. 2006) and CsrA, that binds to the HilD leader transcript, thus blocking its translation (Martínez et al. 2011). In vitro this negative regulation is counteracted by a complex regulatory cascade in which the BarA/SirA two-component system directly activates the expression of two small RNAs, CsrB and CsrC by binding to their regulatory regions. These RNAs bind to and sequester CsrA, which counteracts HilD translational repression. HilD activated, binds to *hilA* and *ssrB* promoters thus counteracting H-NS negative effect and, therefore triggering expression of both islands (Bustamante et al. 2008; Martínez et al. 2011). This regulatory cascade was demonstrated in vitro when *Salmonella* was cultured in LB (Luria-Bertani) medium and it was suggested to be important for the intestinal salmonellosis (Martínez et al. 2011). Even if this cascade has been studied in vitro, there was no evidence of its activation in vivo when *Salmonella* is in hostile conditions, such as the intestinal environment.

To study salmonellosis, several animal models such as the murine, bovine, avian, among others have been employed (Giacomodonato et al. 2022; Nunes et al. 2010; Troxell et al. 2015). It had been reported that the avian model allows the study of both intestinal and systemic salmonellosis (Eade et al. 2018; Withanage et al. 2005) and we previously proved the suitability of chickens for the study of *S.* Typhimurium colonization, dissemination and tissue lesions production (Pico-Rodríguez et al. 2023).

We believe that the cascade formed by *sirA*, *csrB/C* and *hilD* functions when *Salmonella* is in cecal lumen, and it could be important to induce the SPI-1 and SPI-2 genes prior to the intestinal invasion. This would explain the intestinal SPI-2 gene expression previously reported (Brown et al. 2005), as well as the SPI-2 role for inducing complete intestinal disease in some animal models (Coburn et al. 2005; Coombes et al. 2005; Pico-Rodríguez et al. 2023). The aim of this study was to determine if *sirA*, *csrB/C* and *hilD* also conforms a regulatory cascade in vivo and if these genes induce the expression of SPI-1 (*hilA*) and SPI-2 (*ssrB*) genes prior to the intestinal invasion of *Salmonella*. In addition, we analyze the individual contribution of these genes in intestinal colonization, systemic dissemination and histopathological lesions during the infection in chickens.

## Methods

### Bacterial strains and growth conditions

We employed the Wild Type (WT) strain of *S.* Typhimurium SL1344 as well as its derivative mutants Δ*sirA*, Δ*csrB/C* and Δ*hilD* (Table 1). Bacterial cultures were grown overnight at 37° C in Luria Bertani (LB) broth medium in an orbital shaking incubator (Incushaker mini; Benchmark) at 200 rpm. The next day these cultures were transferred to another culture and were grown for 5–6 h under the same conditions; subsequently the cultures were concentrated by centrifugation. When necessary, cultures were supplemented with streptomycin (100 µg ml^− 1^) or kanamycin (20 µg ml^− 1^).

**Table 1:** Strains and primers used in this work

Strains		
Strain name	Description	Reference
*S*. Typhimurium WT	Wild Type SL1344, Str^R^	Hoiseth and Stocker 1981
*S*. Typhimurium Δ*sirA*	SL1344 derivative mutant, Str^R^ Km^R^	Martínez et al. 2011
*S*. Typhimurium Δ*csrB/C*	SL1344 derivative mutant, Str^R^ Km^R^	Martínez et al. 2011
*S*. Typhimurium Δ*hilD*	SL1344 derivative mutant, Str^R^ Km^R^	Martínez et al. 2011
**Primers**		
**Primer name**	**Sequence (5’3’)**	**Reference**
SirAF SirAR	GGA TAC GAC GCA TTC TTG AAG GGA TAC GAC GCA TTC TTG AAG	Martínez et al. 2011
CsrB F CsrB R	GGA TGA AGC AAA GTG GAA AGC AGC TTC TTC CTG AAG CGT CC	Martínez et al. 2011
HilD F HilD R	GCA GGT AGT TAA CGT GAC GC TTG CTG CTC GTT TGG GAT AAG	Martínez et al. 2011
HilA F HilA R	TAC GAC GTA TTC TGT CGG AAG GTA GGT TGC GGC GCT GGC	Martínez et al. 2011
SsrB F SsrB R	GGG TAT ACC AAT CAT GGG ATC CAC AGT TAA GTA ACT CTG TCA C	This work
DnaK F DnaK R	CGT CAG GCA ACC AAA GAT GC CGC GAT AGT ACG GTT GCC G	Martínez et al. 2011

### Experimental animals

1-day-old specific-pathogen-free (SPF) chickens were acquired from ALPES (Mexico). Chickens were kept in isolation units at 30 °C which was progressively reduced to 25 °C until they were 1-week-old. They had free access to water and commercial food.

### Infection experiments

For the infections, groups of 15 chickens were inoculated orally at one day of age with 10^10^ colony-forming units (CFUs) of the WT strain or the Δ*sirA*, Δ*csrB/C* and Δ*hilD* mutants. As we previously reported, for *post mortem* analysis five chickens from the 1-day old infected groups were killed at 24, 48 and 72 h post infection (hpi), respectively; while five chickens from the 1-week old infected groups were killed at 1, 3, and 7 days post infection, respectively (Pico-Rodríguez et al. 2023). Samples of liver and ceca were aseptically collected during *postmortem* analysis, and they were processed to obtain CFU counts, as well as perform histopathological and immunohistochemical analysis.

For the gene expression analysis, groups of 15 1-day-old chickens were inoculated with the WT strain or Δ*sirA*, Δ*csrB/C* and Δ*hilD* mutants. After 120 min post inoculation, chickens were killed and subjected to *postmortem* analysis.

In all cases an additional group was inoculated with PBS as a negative control.

### Determination of colony-forming-units (CFUs)

During the necropsies, cecum and liver samples were obtained in sterile bags and refrigerated. Organs were macerated and homogenized in sterile 1x PBS, subsequently serial ten-fold dilutions were made and plated on LB and McConkey agar with the addition of 100 µg ml^−^^1^ streptomycin for the WT strain, and 100 µg ml^− 1^ kanamycin for mutant strains and incubated at 37° C for 24 h.

### Histopathological analysis

At *postmortem* analysis, cecum and liver samples were placed in 10% neutral buffered formalin for 24 h. Then, fragments of the organs were embedded in paraffin, sectioned and stained with hematoxylin and eosin (H E). To evaluate them, pathological scores were determined as we reported previously, taking in account apoptotic bodies, vacuolar degeneration and heterophilic infiltrate in the cecal samples as well as necrotic foci and inflammatory infiltrate for the liver samples (Pico-Rodríguez et al. 2023).

### Immunohistochemistry

To immunodetect the WT and mutant strains along the infection, we used a polyclonal anti-*Salmonella* Typhimurium primary antibody (BIOSS) in cecum and liver samples processed as we reported previously (Pico-Rodríguez et al. 2023).

### Quantitative real-time RT-PCR (q-RT-PCR) assays

Total RNA was extracted from the cecal content of chickens that were euthanized at 120 min postinoculation using the commercial RNeasy Plus Mini kit (Qiagen). 2 µg of RNA from each sample were incubated with 2 µl of DNase I (Invitrogen) according to the manufacturer’s instructions to remove chromosomal DNA. To synthesize cDNA, we used the Revert Aid H Minus First Strand cDNA Synthesis kit (Thermo Scientific). Each reaction contained 0.5 µg of each DNase-treated-RNA and 5 pmol of reverse primers for *sirA*, *csrB*, *hilD*, *hilA*, *ssrB*, and *dnaK* (Table 1). qRT-PCR reactions were performed in a final volume of 20 µl containing 5 ng µl^− 1^ of cDNA, 10 µl of commercial SensiFAST SYBR No-ROX mix (Bioline) and 5 pmol of the primer pairs for *sirA*, *csrB*, *hilD*, *hilA*, *ssrB*, and *dnaK* (Table 1). Reaction conditions were 10 min at 95 °C, and 40 cycles at 95 °C for 15 s and 60 °C for 60 s. The *dnaK* mRNA levels were used as an internal control to normalize the results obtained for the mRNA of the different genes. The 2^−ΔΔ*C*^_T_ method described by Livak and Schmittgen (2001) was used to analyze data (Livak and Schmittgen 2001). All qRT-PCR reactions for each gene were performed 3 times independently and by triplicate each time.

### Statistical analysis

Histopathological scores were compared using the nonparametric Kruskall-Wallis test. Bacterial counts were compared using analysis of variance (ANOVA) and Tukey’s multiple comparison post tests. qRT-PCR data obtained by 2^−ΔΔ*C*^_T_ method were compared using analysis of variance (ANOVA). All analyzes were performed using SPSS Statistics 19 software.

## Results

### *sirA*, *csrB/C* and *hilD* contribute to the cecum colonization and liver dissemination during *S.* Typhimurium infection of 1-day-old and 1-week-old chickens

To determine the role of *sirA*, *csrB/C* and *hilD* in intestinal colonization and systemic dissemination in vivo, we performed infections in 1-day- and 1-week-old SPF chickens with a WT strain of *S.* Typhimurium and the Δ*sirA*, Δ*csrB/C* and Δ*hilD* mutants to quantify the CFU recovered from the cecum and liver at different times.

**Fig. 1:**
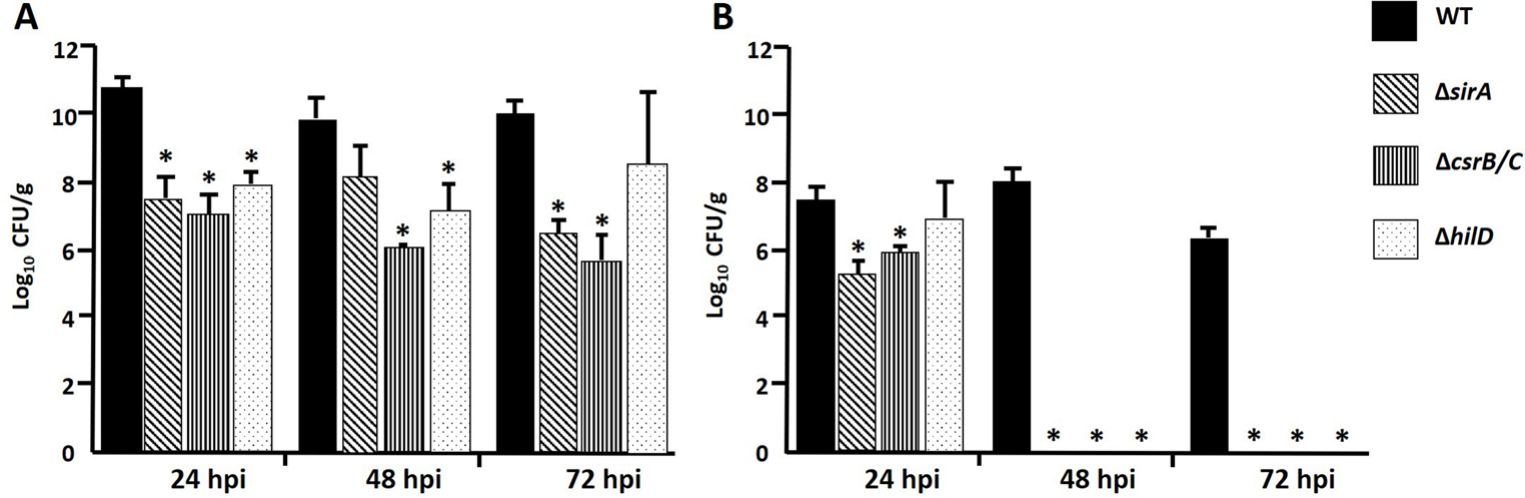
Recovery of *S.* Typhimurium Wild Type (WT) SL1344, Δ*sirA*, Δ*csrB/C* and Δ*hilD* strains from cecum (**A**) and liver (**B**) samples from oral inoculation of 1-day-old chickens. Bacterial counts were made on LB agar and are expressed in CFU/g. Asterisks indicate significant differences between a mutant strain and WT strain (*P < 0.05)

In 1-day-old birds, the WT strain was recovered in constant amounts (10^10^) from the cecum at 24, 48 and 72 hpi. On the other hand, the three mutant strains were recovered in lower amounts (10^6^-10^8^) than the WT strain at all times analyzed (Fig. 1A). Regarding the liver, WT strain was also recovered constantly at the times analyzed, although, in a smaller amount compared to the cecum (10^8^ vs. 10^10^). As for the mutants, they only were recovered at 24 hpi and none of them was recovered at 48 or 72 hpi (Fig. 1B).

**Fig. 2:**
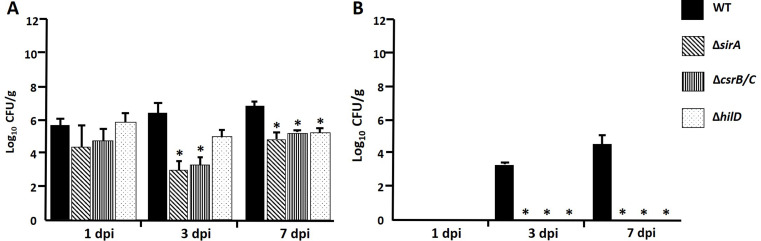
Recovery of *S.* Typhimurium Wild Type (WT) SL1344, Δ*sirA*, Δ*csrB/C* and Δ*hilD* strains from cecum (**A**) and liver (**B**) samples from oral inoculation of 1-week-old chickens. Bacterial counts were made on LB agar and are expressed in CFU/g. Asterisks indicate significant differences between a mutant strain and WT strain (*P < 0.05)

On the other hand, in 1-week-old chickens WT strain was also constantly recovered from the cecum along the infection (10^6^), but the amounts were significatively lower to those observed in ceca from 1-day-old chicken (10^6^ vs. 10^10^) (Fig. 2A), which indicates that the bacteria colonize better newly hatched chickens maybe because their poor immune responses and microbiological conditions. In contrast, mutant strains were recovered in significatively lower amounts at 3 and 7 dpi. In the liver, WT strain was recovered from 3 to 7 dpi samples and in lower quantities (10^4^) than those recovered from the cecum. Interestingly, mutant strains were not recovered from the liver at any time analyzed (Fig. 2B).

Taken together, these results show SirA, CsrB/C and HilD individually contribute during in vivo infection when *S.* Typhimurium colonizes the ceca and disseminates to the liver in 1-day-old and 1-week-old chickens.

### *sirA*, *csrB/C* and *hilD* are necessary to induce histopathological changes in 1-day-old and 1-week-old chickens infected with *S.* Typhimurium

**Fig. 3:**
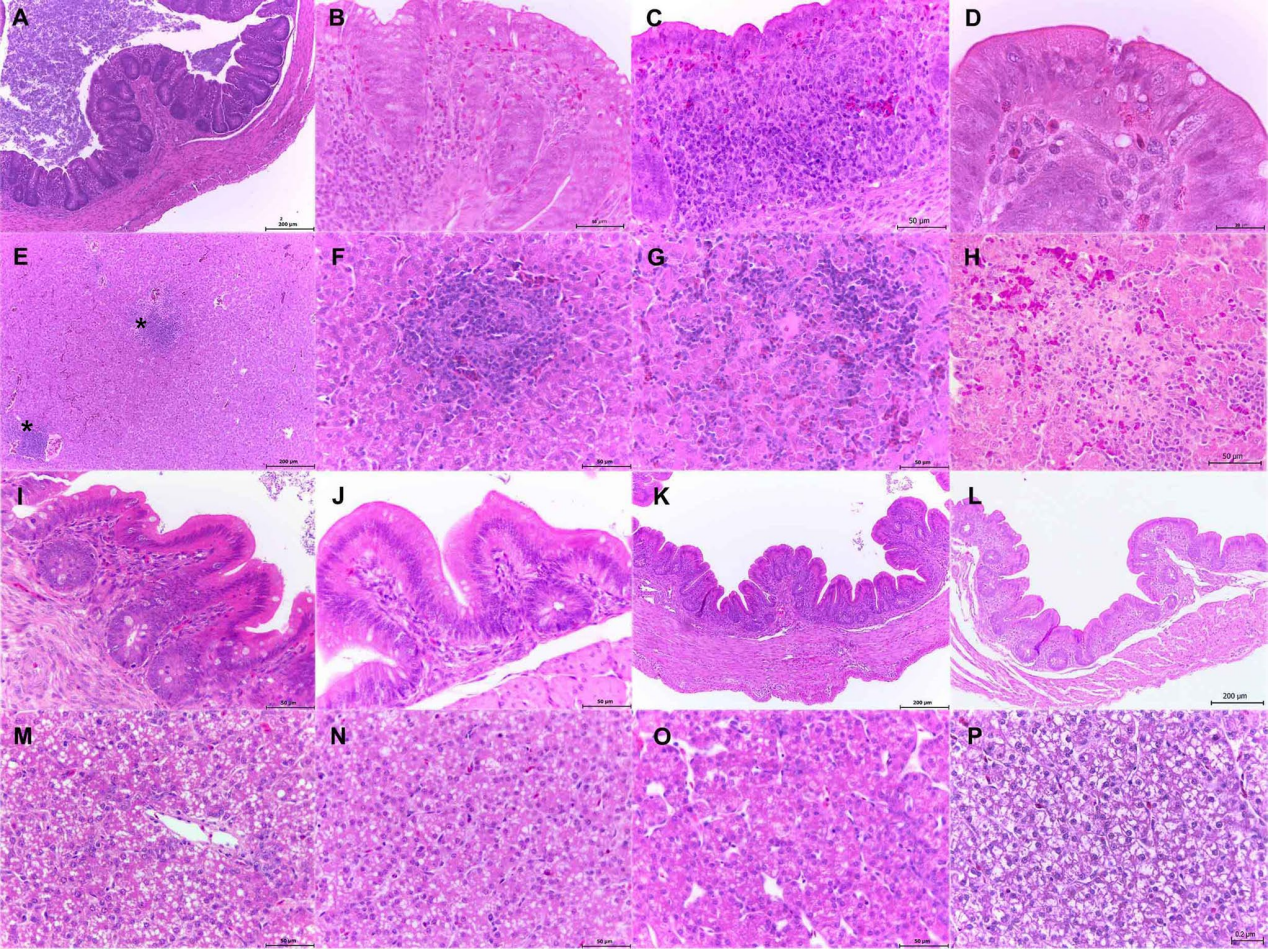
Photomicrographs of cecum and liver sections stained with H and E from 1-day-old chickens inoculated with *S.* Typhimurium WT strain (**A**-**H**) or its derivative mutants Δ*sirA* (**I**, **M**), Δ*csrB/C* (**J**, **N**) and Δ*hilD* (**K**, **O**). WT, cecum: presence of bacilli in the intestinal lumen and crypts, 10x (**A**); vacuolar degeneration and heterophilic infiltrate expanding the lamina propria, 40x (**B**,**C**); epithelial hyperplasia and necrosis, 100x (**D**). WT, liver: lymphoid cumulus (asterisks), 10x (**E**); necrotic areas and lymphoid cumulus, 40x (**F**-**H**). Mutant strains, cecum: Δ*sirA*, 40x (**I**); Δ*csrB/C*, 40x (**J**); Δ*hilD*, 10x (**K**). Mutant strains, liver: Δ*sirA*, 40x (**M**); Δ*csrB/C*, 40x (**N**); Δ*hilD*, 40x (**O**). Controls with no lesions: cecum, 10x (**L**) and liver, 40x (**P**)

We evaluated sections of cecum and liver recovered from 1-day-old infected chickens at 24, 48 and 72 hpi and 1-week-old infected chickens at 1, 3 and 7 dpi. In the sections of ceca from 1-day-old chickens infected with the WT strain we observed large amounts of intraluminal bacteria (Fig. 3A) as well as several morphological changes that increased as the infection progressed. These included vacuolar degeneration and inflammatory infiltrate which expanded the lamina propria (Fig. 3B and C). As the infection progressed we also observed areas of epithelial hyperplasia and necrosis (Fig. 3D). In the liver, several areas of inflammatory infiltrate, hemorrhages and necrosis were observed along the infection (Fig. 3E-H). In contrast, those ceca from chickens infected with the mutants *sirA*, *csrBC* and *hilD*, showed significantly lower amounts of intraluminal bacteria, as well as scant or no lesions (Fig. 3I-K). No lesions were observed in the liver infected with mutant strains (Fig. 3M-O). Those chickens used as control did not show any lesions either (Fig. 3L and P).

**Fig. 4:**
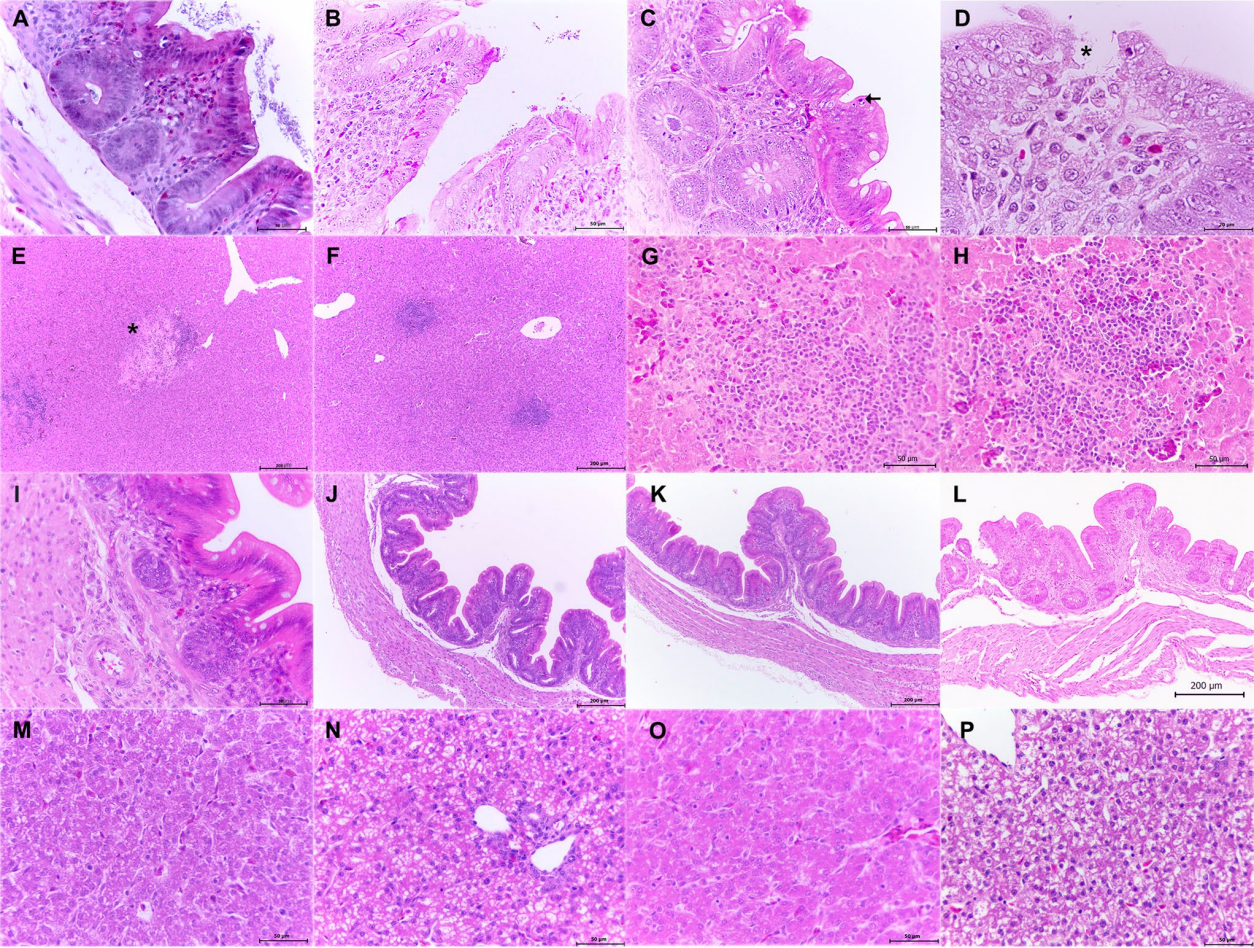
Photomicrographs of cecum and liver sections stained with H and E from 1-week-old chickens inoculated with *S.* Typhimurium WT strain (**A**-**H**) or its derivative mutants Δ*sirA* (**I**, **M**), Δ*csrB/C* (**J**,**N**) and Δ*hilD* (**K**,**O**). WT, cecum: presence of bacilli in the intestinal lumen and crypts, 40x (**A**); exocytosis, 40x (**B**); apoptotic bodies (arrow), 40x (**C**); erosions and ulcers (asterisk) associated with necrotic foci and luminal bacilli, 100x (**D**). WT, liver: necrosis (asterisk), 10x (**E**); cumulus of lymphocytes, 10x (**F**); necrosis and hemorrhage, 40x (**G**,**H**). Mutant strains, cecum: Δ*sirA*, 40x (**I**); Δ*csrB/C*, 10x (**J**); Δ*hilD*, 10x (**K**). Mutant strains, liver: Δ*sirA*, 40x (**M**); Δ*csrB/C*, 40x (**N**); Δ*hilD*, 40x (**O**). Controls with no lesions: cecum, 10x (**L**) and liver, 40x (**P**)

In 1-week-old chickens infected with the WT strain, the ceca showed variable intraluminal and intracryptal bacteria (Fig. 4A), exocytosis (Fig. 4B) and apoptotic bodies (Fig. 4C). Some areas of erosions and ulcers associated with necrotic foci and luminal bacilli were observed starting at 3 dpi (Fig. 4D). In the liver, aggregates of lymphocytes, necrosis and hemorrhage were visualized (Fig. 4E-H). These lesions were more severe than those observed in 1-day-old- chickens.

Compared to the WT strain, in 1-week-old chickens, Δ*sirA*, Δ*csrB/C*, and Δ*hilD* mutants produced fewer lesions in the cecum (Fig. 4I-K) and they did not produce lesions in the liver (Fig. 4M-O). As expected, no lesions were found in the control samples (Fig. 4L and P).

These findings concur with the bacterial counts (Figs. 1 and 2), in which the mutant strains were not recovered or were recovered in lower amounts than the WT. Taken together, these results demonstrate that *sirA*, *csrB/C* and *hilD* are necessary for *S.* Typhimurium to produce lesions in the cecum and liver of 1-day- and 1-week-old infected chickens.

### *sirA*, *csrB/C* and *hilD* mutant strains of *S.* Typhimurium are less immunolocated through the infection in comparison with the WT strain

**Fig. 5:**
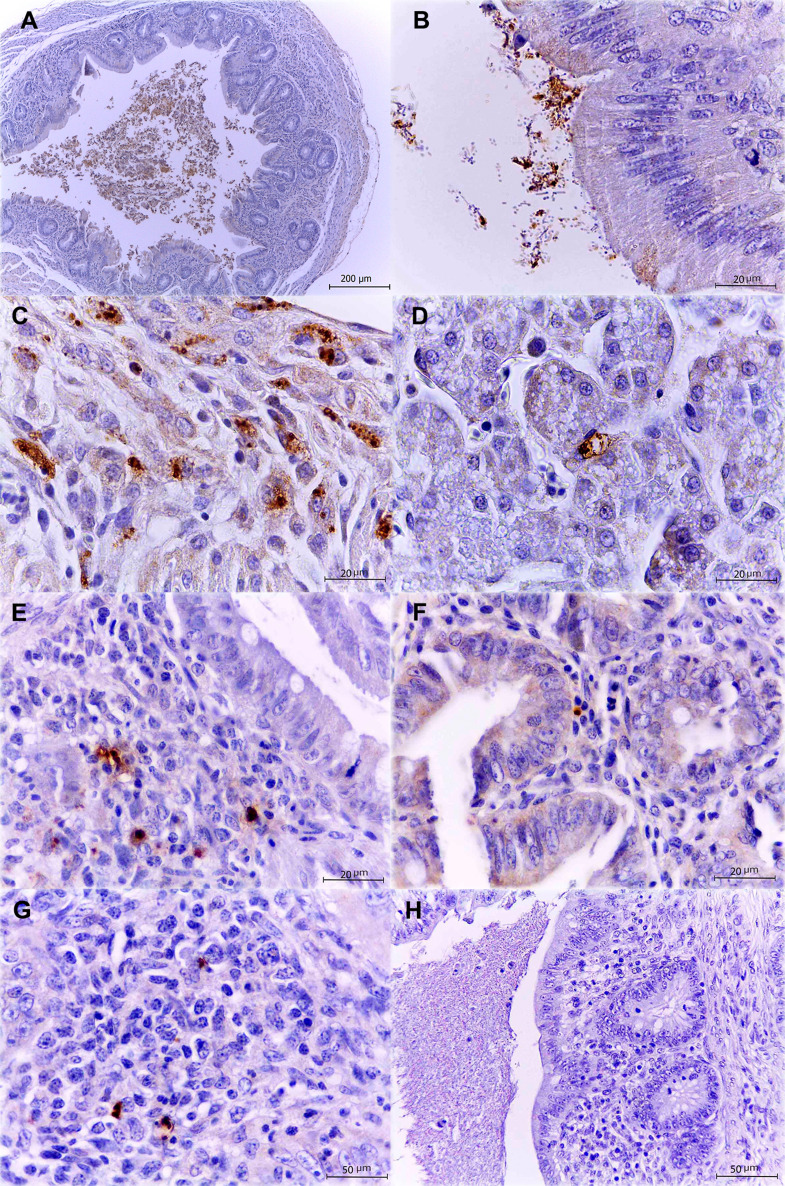
Detection of *S.* Typhimurium by immunohistochemistry with polyclonal anti-*Salmonella* Typhimurium antibody. Bacterial clusters can be seen as brown marks located in different areas of the tissues. WT: cecal lumen, 4x (**A**); bacteria adhered to the intestinal epithelium, 100x (**B**); bacteria in lamina propria, as well as inside enterocytes and some macrophages, 100x (**C**); bacteria inside hepatocytes and areas of hepatic necrosis, 100x (**D**). Mutant strains, cecum: Δ*sirA*, 100x (**E**), Δ*csrB/C*, 100x (**F**) and Δ*hilD*, 40x (**G**) mutant strains. Control group, 40x (**H**)

In order to corroborate our bacterial counts analysis and histopathological results we detected our strains in the tissues using the immunohistochemistry technique. In both infections, WT strain was progressively located as long as the infection progressed. First, it was located in the cecal lumen (Fig. 5A), intestinal crypts and adhered to the epithelium (Fig. 5B); then it was also located multifocally in the lamina propria, as well as inside enterocytes and some macrophages (Fig. 5C). At final stages of 1-day- and 1-week-old infected chickens it was observed inside hepatocytes and areas of hepatic necrosis (Fig. 5D).

In the ceca inoculated with the Δ*sirA* (Fig. 5E), Δ*csrB/C* (Fig. 5F) or Δ*hilD* mutants (Fig. 5G), sporadic and diffuse bacterial foci were detected in both 1-day-old and 1-week-old chickens. These positive labeling foci were detected in the lamina propria and were not associated with the presence of inflammatory infiltrate. In the liver, any mutant was detected along the infections.

### SirA, CsrB and HilD act as a cascade in vivo and control expression of *hilA* and *ssrB* when *Salmonella* is in intestinal lumen avian

In vitro (LB medium), the system SirA/BarA induces expression of CsrB and CsrC that derepress translation of HilD, which once activated, induces the expression of HilA and SsrB, central regulators of SPI-1 and SPI-2. Considering that *sirA*, *csrB/C* and *hilD* contribute to intestinal colonization, hepatic dissemination and lesion production in our avian model, we wanted to assess if these genes form also in vivo a regulatory cascade that induces the expression of *ssrB* and *hilD* prior to the *Salmonella* invasion to the intestine.

The expression of *sirA*, *csrB*, *hilD*, *hilA* and *ssrB* was analyzed in the WT and Δ*sirA*, Δ*csrB/C* and Δ*hilD* strains, by real-time PCR assays using RNA extracted from the cecal content at 120 min post inoculation, moment in which the bacteria is in the intestinal lumen, prior to cecum invasion (Ferrando et al. 1987; Blajman et al. 2017).

**Fig. 6:**
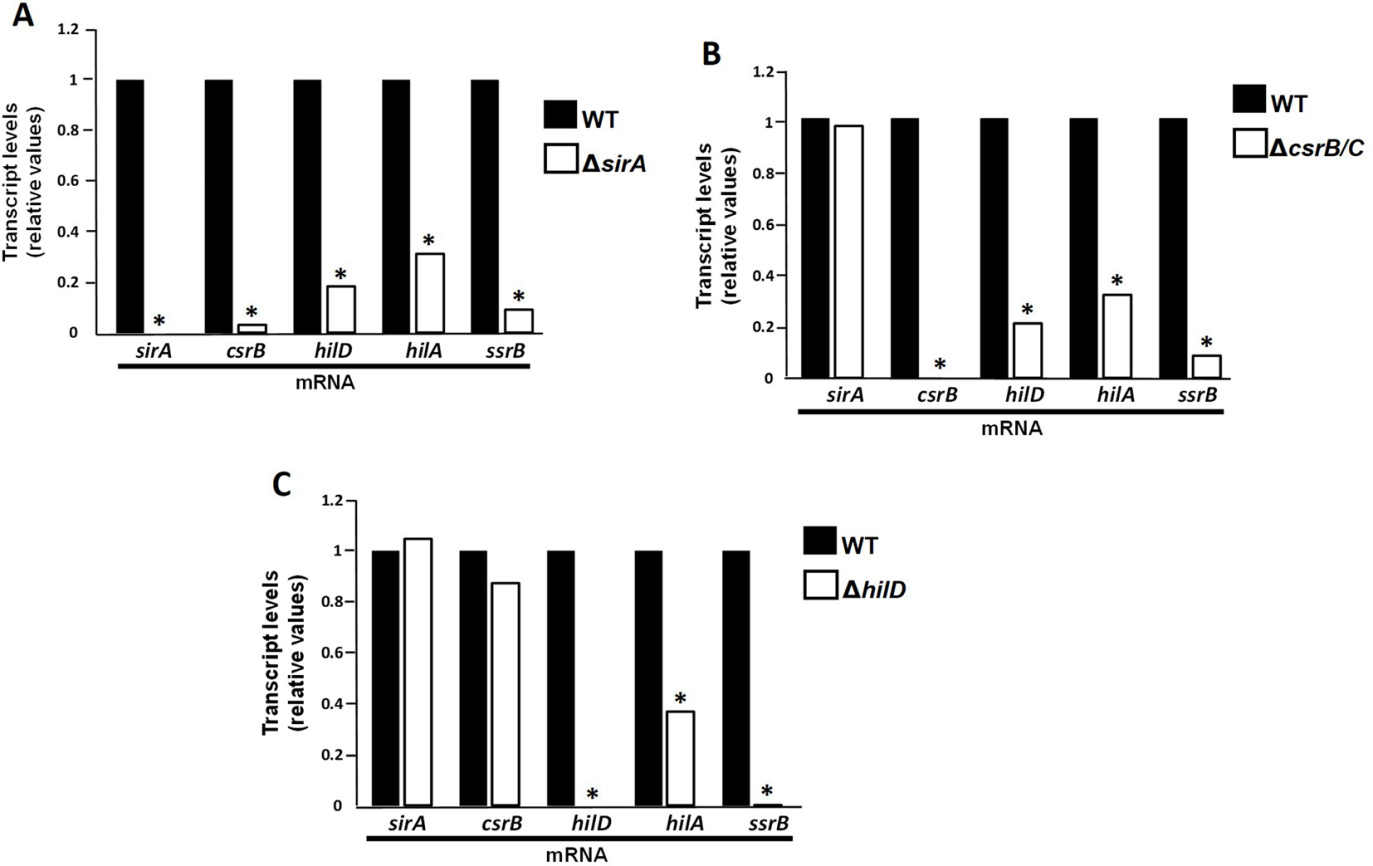
Relative expression of *sirA*, *csrB*, *hilD*, *hilA* and *ssrB* mRNA using bacterial RNA extracted from the cecal content of 1-week-old chickens inoculated with a WT strain and a Δ*sirA* (**A**), Δ*csrB/C* (**B**) and Δ*hilD* (**C**) mutants of *S.* Typhimurium. Samples were taken 120 min after oral inoculation. Asterisks indicate significant differences between a mutant strain and WT strain (*P < 0.05)

First, we examined expression of these genes in a Δ*sirA* mutant strain. As expected, the expression levels of *hilA*, *ssrB*, *hilD* and *csrB* were decreased in this mutant compared to their expression in the WT strain (Fig. 6A), which clearly shows that SirA controls the expression of these genes in vivo.

Next, we analyzed the expression of these genes using a Δ*csrB/C* double mutant to avoid the compensatory effect of the single mutants reported previously (Fortune et al. 2006; Weilbacher et al. 2003). Compared to the WT strain, the expression of *sirA* was not affected, demonstrating that the CsrB/C does not control the expression of *sirA*. In contrast, the expression of *hilA*, *ssrB*, and *hilD* was affected, which shows that their expression is controlled by CsrB/C (Fig. 6B).

Finally, in a Δ*hilD* mutant, only the expression of *hilA* and *ssrB* was affected, being more drastic in *ssrB*. In contrast, the expression of *sirA* and *csrB* was not affected in this mutant (Fig. 6C). This result indicates that HilD controls the expression of *hilA* and *ssrB*, but not *sirA* and *csrB*.

Together, our results show that these genes act in vivo in a cascade fashion, in which SirA induces the expression of *csrB* which in turn induces the expression of HilD to finally induce the expression of *hilA* and *ssrB*, and consequently, of SPI-1 and SPI-2. These results also confirm that this regulatory cascade is induced when *Salmonella* is in the intestinal lumen of chickens, prior the intestinal invasion.

## Discussion

*Salmonella* Typhimurium is a pathogen that requires expression of the SPI-1 genes to invade host cells, whereas SP1-2 genes are necessary to survive and proliferate in the intestinal cells of a wide variety of mammals and birds (Tanner and Kingsley 2018). In vitro (LB medium), the two-component system SirA/BarA induces expression of *csrB* and *csrC*, two small non-coding RNAs that sequester the regulator CsrA, which counteracts its negative effect on the transcript of HilD, encoded in SPI-1. HilD then induces the expression of *hilA* and *ssrB*, central regulators of SPI-1 and SPI-2, respectively, and, consequently, of the rest of the genes in both islands (Martínez et al. 2011).

Using an avian model, we demonstrate that *sirA*, *csrB/C* and *hilD* act in a cascade also in vivo and prior to intestinal invasion to activate the expression of *hilA* and *ssrB*, central regulators of SPI-1 and 2, respectively. Furthermore, we show that these genes contribute to intestinal colonization, systemic dissemination, and lesion production.

In 1-day-old chickens, *sirA*, *csrB/C* or *hilD* mutants colonized cecum in lower amounts than the WT strain along the infection, whereas they were recovered from the liver only at 24 hpi. In 1-week-old chickens, mutant strains were recovered from the cecum in lower quantities starting at 3 dpi and none grew in liver at any time, compared to the WT strain.

Individual effect of depleting SirA, CsrB, CsrC and HilD has not been widely studied in animal models, but it has been reported that their absence affects cell invasion or intestinal inflammatory response in cell culture or bovine ligated ileal loops (Ahmer et al. 1999; Altier et al. 2000; Banda et al. 2018; Fortune et al. 2006; Hung et al. 2019).

In this study, we observed that in 1-week-old chickens, *hilD* mutant was recovered significantly less at 7 dpi, whereas none *hilD* mutant was found in liver samples. Similarly, Eade et al. showed that in 4-days-old chickens inoculated with *Salmonella* Enteritidis, the WT strain and a *hilD* mutant were recovered from cecum in similar amounts at 2 dpi, but at 9 dpi, the absence of *hilD* impaired the ability of *Salmonella* to colonize the cecum (Eade et al. 2018). Futhermore, in agreement with our results, they found that the *hilD* mutant was drastically affected to disseminate systemically (Eade et al. 2018). Although there are differences in bacterial strains and experimental procedures in both studies, it is clear that the absence of HilD has greater impact at later stages of intestinal colonization.

Here we show that the absence of *sirA* affects both the colonization and systemic dissemination. Ahmer et al. evaluated the role of SirA in two animal models. In bovine ligated ileal loops, they found that a *sirA* mutant reduced the inflammatory response, which concurs with our CFU analysis. On the other hand, in mice they reported that SirA has little or no effect in the virulence of *Salmonella* Typhimurium (Ahmer et al. 1999). This difference could be attributed to the fact that mice have been reported as a convenient model for systemic disease but no for the study of enteropathogenesis (Ahmer et al. 1999) whereas chickens have been shown to be a suitable model to study both the systemic and intestinal salmonellosis (Withanage et al. 2004, 2005). It has been previously suggested that SirA is required when *Salmonella* is in the intestinal lumen (Martínez et al. 2011), therefore in order to evaluate that suggested role of SirA, we employed chickens in this study.

Individual role of SirA, CsrB, CsrC and HilD in vivo had not been widely studied previously. Taken together, our results show that these regulators are involved in survival and proliferation in cecum and liver in 1-day-old and 1-week-old chickens.

As we previously reported, 1-day- and 1-week-old chickens infected with the WT strain showed morphological changes in both cecum and liver, such as vacuolar degeneration, apoptotic bodies in the epithelial tissue, heterophilic infiltrate and necrosis foci. Intraluminal and intracryptal bacteria were also observed in both groups of chickens.

Chickens infected with the Δ*sirA*, Δ*csrB/C* and Δ*hilD* mutants showed significantly lower amounts of intraluminal bacteria, as well as scant or no lesions in both the cecum and the liver. This result shows that SirA, CsrB/C and HilD are essential for *S*. Typhimurium to produce lesions in both organs. Few studies have evaluated the role of these genes to produce morphological changes in tissues during the infection. Ahmer et al. demonstrated that *hilA* and *sirA* are required for accumulation of fluid in the intestine, as well as the neutrophil migration in a bovine ligated ileal loop model. Taken together, our results and those reported by Ahmer confirm the role of SirA in the pathogenesis of *S*. Typhimurium. Additionally, Eade et al. reported that a Δ*hilD* mutant of *S*. Enteritidis produce less injuries and inflammatory response in ceca, than a WT strain (Eade et al. 2018), which agrees with our results with the Δ*hilD* mutant of *S*. Typhimurium. These findings highlight the essential role of HilD to produce lesions during a *Salmonella* infection in chickens.

Concerning CsrB and CsrC it has been only evaluated their role in cell culture invasion but no their contribution to produce tissue lesions during the infection. Therefore, to our knowledge this is the first study that demonstrate the crucial role of both CsrB and CsrC in the pathogenesis of *S*. Typhimurium in chickens.

Previously, we used IHC to follow the infection of *S*. Typhimurium in both 1-day-old and 1-week-old chickens (Pico-Rodríguez et al. 2023). Here we used the IHC to also track Δ*sirA*, Δ*csrBC* and Δ*hilD* mutants. Only scant foci of the three mutants were immunolocated in the cecum through the infection. Our results show for the first time the immunolocation of these strains and reveal that the absence of *sirA*, *hilD* or *csrBC* impaired the bacteria to colonize the cecum and disseminate to the liver, which correlates with the absence of lesions in these organs.

All together, our results demonstrate the role of SirA, CsrBC and HilD in cecal colonization, systemic dissemination and injuries production during a *S*. Typhimurium in vivo chickens’ infection.

Previously, it had been demonstrated in vitro that *sirA*, *csrB* and *hilD* form a regulatory cascade that controls SPI-1 and SPI-2 expression (Martínez et al. 2011). In this study we demonstrated that these genes also act in a cascade fashion to induce the expression of both islands in vivo during the infection and they are expressed since *Salmonella* reaches the cecal lumen and prior to intestinal invasion.

We analyzed the expression of *sirA csrB*, *hilD*, *hilA* and *ssrB* in samples of cecal contents obtained 120 min postinoculation, as this is the estimated time for a bacterium to reach the intestinal lumen in chickens (Blajman et al. 2017; Ferrando et al. 1987). First, we observed that the expression of *csrB*, *hilD*, *hilA* and *ssrB* was decreased in the Δ*sirA* strain compared to the WT strain, demonstrating that SirA controls the expression of these genes. This result coincides with previous studies where the in vitro expression of SsrB, *hilA* and *hilD* significantly decreased in a *S.* Typhimurium lacking SirA (Fortune et al. 2006; Johnston et al. 1996; Martínez et al. 2011).

In the Δ*csrB/C* double mutant, expression of *hilD*, *hilA* and *ssrB* decreased, but not that of *sirA*, confirming that *csrB* is downstream of SirA in the regulatory cascade *in vivo.* In agreement, Fortune et al. had reported that the expression of *hilA* is decreased in a Δ*csrB/C* double mutant and Martínez et al. demonstrated that in a *Salmonella* Δ*csrB/C* mutant grown in LB medium, *hilD*, *hilA* and *ssrB* expression was drastically affected. In addition, our results demonstrate that CsrB/C system control the expression of *hilD*, *hilA* and *ssrB* also in vivo, when *Salmonella* is in the intestinal lumen.

Finally, we demonstrate that HilD also controls in vivo the expression of *hilA* and *ssrB* but not that of *sirA* and *csrB*, confirming that HilD acts downstream SirA, CsrB and CsrC to regulate *hilA* and *ssrB*, as it had been previously reported when *S.* Typhimurium grows in LB medium (Martínez et al. 2011).

Previously, Brown et al. showed the in vivo induction of SPI-2 genes in the intestinal lumen, but the mechanism was unknown and Martínez et al. showed that SirA, CsrB/C and HilD conforms a cascade that in vitro induces SPI-1 and SPI-2 genes (Brown et al. 2005; Martínez et al. 2011). Our results assemble those previously reported, confirming that SirA, CsrB/C and HilD also conform in vivo a regulatory cascade to control *hilA* and *ssrB* expression and this cascade could be the responsible of the SPI-2 expression reported by Brown in the intestinal lumen. It is possible that intraluminal induction of SPI-2 genes allows *S.* Typhimurium to initiate transition to the hostile intracellular environment and thus optimally coordinate survival and proliferation mechanisms.

Diverse and complex physicochemical signals have been reported to be present in the intestinal environment; these signals would be sensed by a myriad of different regulators and therefore all they could drive the expression of SPI-1 and SPI-2 genes (Altier 2005; Azimi et al. 2020; Fass and Groisman 2009; Lou et al. 2019). It has been reported that HilE is required for activation of SPI-1 gene expression by acetate independently of BarA/SirA, which normally also induces SPI-1 gene expression in response to acetate (Hamed et al. 2021). Propanediol and ethanolamine, which are nonfermentable carbon compounds that are metabolized in the lumen of the inflamed intestine, induce the activation of *pdu* and *eut*, and it has been reported that SirA-CsrB/CsrC-CsrA regulatory cascade controls the expression of these genes (Nava-Galeana et al. 2023). Lysophosphatidylcholine which is released in *Salmonella*-infected cells following caspase-1 activation, promotes the expression of HilA (Shivcharan et al. 2018).

This study shows that SirA, CsrB/CsrC and HilD genes are conforming a regulatory cascade activated in chicken intestinal lumen to induce the expression of HilA and SsrAB. Furthermore, we demonstrated that *S*. Typhimurium requires these genes for cecal colonization, systemic dissemination and production of lesions during the chicken infection.

The expression of this complex regulatory cascade in the intestinal lumen suggests that the successful transition of this pathogen from the extracellular to the intracellular medium depends largely on different regulators that could respond to different environmental signals and consequently, act to control the expression of numerous genes that enable *Salmonella* to stablish a successful infection.

## Data Availability

No datasets were generated or analysed during the current study.
